# Effect of motivational interviewing on treatment adherence and self‐efficacy of adolescents with asthma: A randomized controlled trial

**DOI:** 10.1002/nop2.1679

**Published:** 2023-03-17

**Authors:** Fatemeh Taheri, Ahmad Nasiri, Somayeh Namdari, Fatemeh Salmani

**Affiliations:** ^1^ Faculty of Nursing and Midwifery Birjand University of Medical Sciences Birjand Iran; ^2^ Department of Nursing Behbahan Faculty of Medical Sciences Behbahan Iran; ^3^ Department of Epidemiology and Biostatistics, Faculty of Health Birjand University of Medical Sciences Birjand Iran

**Keywords:** adolescents, asthma, motivational interviewing, self‐efficacy, treatment adherence

## Abstract

**Aims:**

This study examined the short‐term effect of motivational interviewing on treatment adherence and self‐efficacy of adolescents with asthma.

**Design:**

The randomized controlled trial.

**Method:**

In this study, 72 adolescents with asthma were recruited and assigned to experimental and control groups randomly. In the experimental group, the motivational interviewing was performed for five weekly sessions lasting 80–90 min. The treatment adherence and self‐efficacy questionnaires were completed before the intervention, 2 weeks and 3 months after the intervention in both groups. Data were analysed by Chi‐Square test, independent samples T‐test, repeated measures of Wilcoxon and generalized estimating equation.

**Results:**

The treatment adherence was found to be significantly higher 2 weeks (*p* = 0.006) and 3 months after the intervention (*p* = 0.04) in the experimental group than the control group. In addition, the degree of self‐efficacy was significantly more in the experimental group 2 weeks (*p* < 0.001) and 3 months later (*p* < 0.001) than the control group. The result of generalized estimating equation showed that the intervention group had an average of 14.44 more self‐efficacy points than the control group (*p* < 0.001). Also, treatment adherence in the intervention group was significantly higher than the control group (*β* = 6.14, *p* = 0.05(.

**Conclusion:**

This study adds to the evidence for the effectiveness of motivational interviewing in treatment of adolescents with asthma.

## 1 INTRODUCTION

Asthma is a chronic allergic disorder affecting more than 300 million people in the world, almost 60% of which are children (Gomes et al., 2017). In Iran, 1.6%–11.26% of children and adolescents suffer from asthma (Faraji et al., 2020). Despite the scientific and technological advances in the treatment of asthma, 260,000 people die each year due to the disease. More than 5 million children and adolescents are victims of asthma worldwide (Zarei et al., 2014). Although asthma is most often diagnosed during childhood, when parents have primary control over disease regulation and they remain an important source of social support, as a child with asthma ages, management responsibilities are often shifted from the parents to the adolescent (Ayala et al., 2009). Many adolescents with asthma have suboptimal disease control despite the availability of effective therapies. For some, poor asthma control will be a consequence of suboptimal self‐management, particularly adherence to treatment (Holley et al., 2019). On the other hand, in relation to asthma, low adherence is one of the most common causes of lack of clinical and functional disease control. In the case of children, when parents have a negative perception of asthma, they may be hesitant in terms of the need for inhaled drugs considering the side effects of drugs. In addition, when they do not have the necessary information about the disease, thus their adherence levels will be even lower (Jentzsch et al., 2017).

In asthma control, psychological, therapeutic and environmental factors are effective including behaviours focusing on the self‐efficacy (Gomes et al., 2017). Self‐efficacy is referred to the individual's self‐confidence in his/her ability to perform a specific behaviour in a specific situation and is one of the important requirements for changing the health behaviours in patients with asthma (Martin et al., 2009). Among adolescents, higher levels of self‐efficacy are associated with using more asthma management strategies and being more adherent makes it an important target for behavioural interventions (Ayala et al., 2009). So, interventions are needed for adolescents with asthma to control the disease with the aim of improving their self‐efficacy and treatment adherence.

## 2 BACKGROUND

The key principle of asthma control is higher self‐efficacy and stronger treatment adherence in patients (Bektas et al., 2021; Jentzsch et al., 2017). According to previous studies, low levels of self‐efficacy in patients with asthma are probably related to the emotional stress, which in turn has direct physiological effects (Martin et al., 2009). Adolescents with high levels of self‐efficacy are more aware of their health status and health problems compared to those with low and moderate levels of self‐efficacy (Bektas et al., 2021).

In addition to self‐efficacy, adherence to treatment is the other factor related to control of the disease. Patients' understanding from illness and treatment plays an important role in adherence, which is often overlooked. The patient's decisions for follow‐up are heavily influenced by his beliefs and attitudes towards illness and treatment (Chiu et al., 2014). A study (Kaplan & Price, 2020) showed that non‐adherence is particularly concerning in adolescents, who have specific age‐related barriers to taking their medication, which can have a disastrous impact on asthma control and subsequent outcomes. Bender reviewed 10 studies who investigated the contract, in which treatment adherence was reported as 19%–53% (Bender, 2016), while adherence less than 50% indicates low adherence (Jentzsch et al., 2017).

Some previous studies reported that adolescents with asthma suffer from a number of problems, including a recent study (Al Kindi et al., 2021) which reported that Children with asthma have more susceptibility for physical and emotional disabilities, and that their academic achievement is more likely to decline. Almost including 36,000 children estimated who miss school every day due to the disease symptoms, putting them at greater risk for poor academic performance and poor intellectual development. Another study (Myers et al., 2018) reported that global pharmaceutical cost of the disease is also more than $ 5 billion a year.

Motivational interviewing is an advisory and patient‐based approach that can be focused on the patients' challenges briefly; it was specially designed to provide incentives for change among the patients who are not ready for change (Borrelli et al., 2007; Fortune et al., 2019). The process involves detection, identification; and solving the doubt (Mirkarimi et al., 2017). The motivational interviewing helps patients to resolve their persistency in behaviour change, and provides an inherent motivation before offering the education. The motivational interviewing consists of some underlying motivational strategies that can be implemented concisely and easily in medical environments (including planning, assessing the motivation and confidence for change and providing medical consultation and health feedback). Reflexive listening is used to help the patients to recognize the duality and increase the resistance (Borrelli et al., 2007). Motivational interviewing can be more appropriate for adolescents because of the promotion of personal control and autonomy in decision making for change, focusing on individual goals and values, and their relationship with the target's behaviour, and willingness to change (Morton et al., 2015).

Despite the recent use of this method in the health systems, for health promotion, especially in the field of education and rehabilitation of mental disorders (Mirkarimi et al., 2017), few studies have been done on patients with asthma, especially adolescents in this field.

The purpose of this study was to examine the short‐term effects of motivational interviewing on adherence and self‐efficacy in adolescents with asthma.

## 3 METHODS

### 3.1 Study design

A randomized clinical controlled trial design was used in this study.

### 3.2 Participants

**FIGURE 1:**
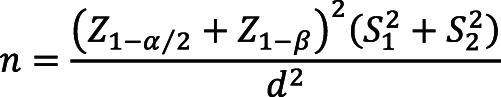
Sample size calculation equation.

Participants included the adolescents with uncontrolled asthma who were referred to paediatric clinics. The inclusion criteria were adolescents aged 10–15; all of them had a history of asthma clinically diagnosed at least 1 year ago as well as using the inhaled corticosteroids under the supervision of the paediatrician. The exclusion criteria were those who were receiving medical treatment for physical and/or mental illnesses and who were absent for more than one session. After preparing the list of adolescents, those who were willing to participate in the study were selected. The sample size was calculated based on the results of a Zarei et al study (Zarei et al., 2014), and with a standard deviation in intervention group of 3.55, and 6.82 in control group and absolute error 4.5, power of 0.90 and confidence level of 95%. The sample size calculation equation (Figure 1) showed that 31 participants were needed for each group. The sample size was increased to 36 considering of 20% attrition rate (72 adolescents with asthma).

**FIGURE 2:**
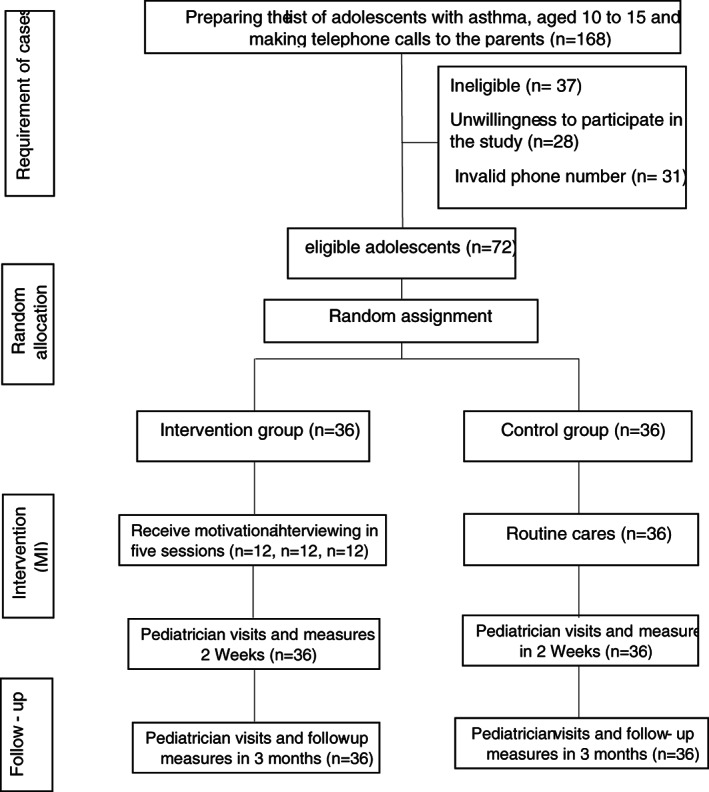
Flow chart of the study participants.

After attending the appointment and obtaining the written informed consent from the parents, adolescents were randomly assigned in experimental and control groups. Figure 2 shows how the study participants were recruited.

### 3.3 Data collection procedures

Demographic information, the asthma self‐efficacy questionnaire (ASQ), adherence self‐report and asthma control questionnaire (Kercsmar et al.) were measured before the intervention for two groups. The intervention group received the motivational interviewing for 5 weeks, and the control group received no treatment, then all the mentioned variables were measured 2 weeks and 3 months after the intervention in both groups.

### 3.4 Ethical considerations

The study was approved by the university research ethics committee (code: IR.BUMS.REC.1395.179). Before the intervention, the adolescents and their parents were given information about the aim of the study and intervention. They also were informed about their rights as participants. Then a written informed consent form was filled out by the parents. In order to privacy protection the data collected from adolescents were coded anonymously.

### 3.5 Control group

The adolescents in the control group just receive regular nursing care. They did not receive psychological or motivational interventions except in special cases.

### 3.6 Intervention group: Motivational interviewing

The experimental group was randomly divided into three subgroups of 12 persons, and motivational interviewing was performed for each group according to the Miller and Rollnic by one of the researchers who had master of psychiatric nursing within 5 weekly sessions of 80–90 min. Parents of the adolescents were present at the meetings.

The trainer explained the principles of motivational interviewing, for example, active listening to express empathy, evoking the counselee's autonomy for decision making, developing contrast between the counselees' intentions, present behaviour, and promoting their self‐efficacy. They were also explained about the duration of the sessions and were informed that they have 80–90 min for each session. In the sessions, patient‐centred communication techniques including open‐ended questions, providing affirmation, empathy, reflective listening and providing summaries were used. Also, adolescents were encouraged to participate actively and provide opinions in the group.

Sessions focused on motivating the adolescent and exploring and resolving ambivalence about asthma medication adherence and disease management behaviours. For this purpose, we measured the independence and motivation of adolescents using worksheets, for example, we asked them to give a score on the motivation regarding adherence to medications and physician's recommendations on a scale of 0–10 (0 = no motivation at all and 10 = completely motivated). Then, with the guidance of the researcher, the adolescents explored the reasons for the answers (Why not chosen a lower grade? Or what would it take to get you up to a higher grade?)

Then, adolescents were encouraged to verbalize potential benefits of following physician's recommendations and taking their medication (and the potential disadvantages of not following physician's recommendations and not taking their medication). Finally, when the adolescents declared their interest in change, they discussed about the change and the pattern of its creation (Table 1).

**TABLE 1:** Conceptual framework of motivational interviewing sessions.

*First session*	Orientation
	The first meeting included the familiarity and initial assessment of the participants, and introduction of the rules and regulations of the group. Then, the assessments were done to determine the level of freedom and independence of individuals in decision‐making, the dimensions of the effect of behaviour, as well as the assessment of commitment, confidence and assurance
*Second Session*	Emotional Exercise
	Adolescents became aware of their feelings about the different dimensions of life influenced by the behaviours related to asthma. Each person identified his/her feelings and shared them in the group.
*Third Session*	Practicing the benefits and harms
	With the help of subjective pretensions, short and long‐term profits and losses of behaviour were identified, and the suggestions were made on how to change the behaviour by describing the alternative exercises. The researcher helped the adolescents in this regard. In addition, the level of adolescent self‐efficacy was discussed through validation and reflection, or more options.
*Fourth Session*	Exercising the Values
	Adolescents identified and prioritized important life values. Then, the practice of ‘matching the values and behaviour’ was performed. The researcher outlined the concept of differences between the values and behaviour using the examples
*Fifth Session*	Final assessment
	Finally, the purpose of the fifth session was identifying the temptation as well as final assessment, which led to the readiness and beginning of the change in the approach of the participants

### 3.7 Measures

#### 3.7.1 General characteristics

To obtain the demographic information, a questionnaire was used including the questions about age, sex, education level, mother's occupation, father's occupation and family income.

#### 3.7.2 Asthma self‐efficacy

To assess the adolescents' self‐efficacy, an ASQ was used that included five aspects of asthma control (five items), dealing with acute asthma attack (six items), regular use of medication (one item), stimuli and environment (four items), and relationship with the physician (four items), and the responses were scored in five scales: at all (score 1), low (score 2), relatively (score 3), high (score 4) and perfectly (score 5) respectively. Its reliability coefficient has been reported in the study by Martin et al. 0.82 (Martin et al., 2009). In Iran, the content validity of the instrument was confirmed by Valizadeh et al and its reliability was calculated by them. Cronbach's alpha for the scale was 0.85 (Valizadeh et al., 2014).

#### 3.7.3 Treatment adherence

To assess treatment adherence, an adherence self‐report form was administered (Celano et al., 1998) According to this form, the adolescents were asked to take notes from the daily dose of medication (number of sprays) during a week. Then, in each of three steps, the dosage determined by the physician was compared with the dose used by the patient, and the percentage of adherence was determined. The reliability of this questionnaire was evaluated and was obtained as 0.88.

#### 3.7.4 Asthma control

Asthma control questionnaire (Kercsmar et al.) was applied consisting of seven dimensions including waking up at night, severity of symptoms during waking up in morning, limitations in doing the activities, respiratory failure, wheezing, the number of spray puffs used, and FEV1 (forced expiratory volume in 1 s) percentage during the last week. Responses were ranked from 0 to 6 in seven scales respectively. A zero score indicates better control of asthma and a score of 6 indicates weak control of asthma. Cronbach's alpha has been reported as 0.85 in the study by Lavoie et al. (Lavoie et al., 2014). The reliability of the asthma control questionnaire (Kercsmar et al.) also obtained by calculating Cronbach's alpha by Dashti et al in Iran. It was equal to 0.89, which indicated the good reliability of this questionnaire (Dashti et al., 2016).

### 3.8 Data collection and analysis

Data were analysed using SPSS software 19.0. First, the demographic variables in the two groups were compared by Chi‐Square and T‐test. Then, the Kolmogorov–Smirnov test was performed to determine the normality of scores. Its results revealed the normal distribution of all scores. Subsequently, the results were analysed by the independent samples T‐test and repeated measures at different stages. Independent samples T‐test was used to compare the mean scores of adherence and self‐efficacy between the intervention and control group. The repeated measures analysis of variance was used to assess the effect of motivational interviewing on adherence and self‐efficacy in patients in each group. Marginal model with Generalized Estimating Equation (GEE) approach applied for controlling of potential confounders. A P‐value of <0.05 was considered as statistically significant.

## 4 RESULTS

### 4.1 Characteristics of the participants

Seventy‐two participants were included in the study. The sample was comprised of 46 males. The demographic characteristics of the participants were not statistically different in terms of age, sex, level of education, father's occupation, mother's occupation and family income between the two groups (*p* > 0.05). Table 2 shows the demographic characteristics of the participants.

**TABLE 2:** Sociodemographic characteristics of study participants.

Variables	Control group		Intervention group		*p* Value
	*N*	Mean (SD) or %	*N*	Mean (SD) or %	
Age, years	36	11.86 (1.91)	36	11.48 (1.68)	0.30
Sex					
Women, %	12	33.3	14	38.9	0.80
Men, %	24	66.7	22	61.1	
Educational level					
Primary, %	23	63.9	25	69.4	0.24
Guidance school, %	0	0.0	2	5.6	
High school, %	13	36.1	9	25.0	
Father's occupation					
Self‐employed, %	23	63.9	24	66.7	0.96
Employee, %	10	27.8	9	25.0	
Teacher, %	3	8.3	3	8.3	
Mother's occupation					
Employed, %	13	36.1	12	33.3	0.80
Housewife, %	23	63.9	24	66.7	
Family income					
<$200, %	22	61.1	23	63.9	0.96
$200–400, %	12	33.3	11	30.6	
>$400, %	2	5.6	2	5.6	

*Note*: Number (percentage) of yes/correct responses.

### 4.2 Self‐efficacy

The mean score of self‐efficacy in all five areas was significantly higher in the experimental group than the control group 2 weeks after the intervention (*p* < 0.001). It is mentioned that asthma self‐efficacy increased in the experimental group compared to the control group. In addition, a 3‐month follow‐up for the study showed that self‐efficacy was significantly better in the experimental group than the control group (*p* < 0.001). This indicates that the motivational interviewing did not lose its effect even 3 months after the intervention. (Table 3).

**TABLE 3:** Between‐ and within‐group comparisons concerning self‐efficacy.

		Pretest mean (SD)	Posttest mean (SD)	Follow‐up mean (SD)	RM	W		
						Pretest ‐ posttest	Pretest ‐ follow‐up	Posttest ‐ follow‐up
TSS	Intervention	53.97 (8.13)	69.97 (8.57)	68.63 (9.06)	<0.001	<0.001	<0.001	0.42
	Control	50.0 (9.54)	49.22 (8.31)	49.44 (8.65)	0.64	1.00	1.00	1.00
	*p* Value	0.06	<0.001	<0.001	‐	‐	‐	‐
DC	Intervention	13.75 (2.73)	17.33 (2.55)	16.69 (2.65)	<0.001	<0.001	<0.001	0.17
	Control	12.19 (3.80)	12.44 (2.98)	12.83 (2.78)	0.29	1.00	0.88	0.70
	*p* Value	0.05	<0.001	<0.001	‐	‐	‐	‐
MAA	Intervention	16.52 (2.77)	21.36 (2.41)	20.36 (2.73)	<0.001	<0.001	<0.001	0.07
	Control	15.36 (3.25)	14.61 (3.06)	15.08 (3.09)	0.47	0.11	1.00	0.32
	*p* Value	0.10	<0.001	<0.001	‐	‐	‐	‐
RDC	Intervention	2.77 (0.70)	4.11 (0.70)	3.86 (0.76)	<0.001	<0.001	<0.001	0.14
	Control	3.00 (0.82)	3.13 (0.59)	2.86 (0.72)	0.09	0.69	0.28	0.04
	*p* Value	0.22	<0.001	<0.001	‐	‐	‐	‐
ME/E	Intervention	10.00 (2.49)	13.30 (3.22)	13.50 (3.06)	<0.001	<0.001	<0.001	1.00
	Control	8.77 (2.25)	8.69 (2.01)	8.38 (2.04)	0.14	1.00	0.44	0.66
	*p* Value	0.03	<0.001	<0.001	‐	‐	‐	‐
DR	Intervention	10.91 (3.14)	13.86 (3.39)	14.22 (3.44)	<0.001	<0.001	<0.001	0.80
	Control	10.66 (2.83)	10.33 (2.58)	10.27 (2.93)	0.31	0.93	0.94	1.00
	*p* Value	0.72	<0.001	<0.001	‐	‐	‐	‐

Abbreviations: DC, disease control; DR, doctor relationship; MAA, managing an acute attack; ME/E, managing emotions/environment; RDC, remembering daily controller; RM, repeated measures; TSS, total summary score; W, Wilcoxon.

### 4.3 Treatment adherence

Treatment adherence was measured by the self‐report adherence form. Before the intervention, the mean of adherence scores did not differ significantly (*p* = 0.39) between the two groups. In other words, the experimental group had a better adherence compared to the control group. The adherence self‐report score also showed a better adherence of the adolescents in the experimental group than the control group 2 weeks (*p* = 0.006) and 3 months after the intervention (*p* = 0.04). (Table 4).

**TABLE 4:** Between‐ and within‐group comparisons concerning treatment adherence.

		Pretest mean (SD)	Posttest mean (SD)	Follow‐up mean (SD)	RM	W		
						Pretest ‐ posttest	Pretest ‐ follow‐up	Posttest ‐ follow‐up
Self‐report	In	40.09 (13.66)	53.97 (14.13)	52.19 (14.25)	<0.001	<0.001	<0.001	<0.001
	Co	43.39 (18.40)	43.14 (17.80)	44.17 (18.25)	0.34	0.91	1.00	0.72
	P	0.39	0.006	0.04	‐	‐	‐	‐

Abbreviations: Co, control; In, iIntervention; RM, rRepeated mMeasures; W, Wilcoxon.

### 4.4 Asthma control

Before the intervention, the mean of treatment adherence scores did not differ significantly in asthma control questionnaire (*p* = 0.62). The asthma control score was significantly lower in the experimental group than the control group 2 weeks (*p* = 0.03) and 3 months (*p* < 0.001) after the intervention. (Table 5).

**TABLE 5:** Between‐ and within‐group comparisons concerning asthma control.

		Pretest mean (SD)	Posttest mean (SD)	Follow‐up mean (SD)	RM	W		
						Pretest ‐ posttest	Pretest ‐ follow‐up	Posttest ‐ follow‐up
Asthma control	In	11.11 (6.35)	6.44 (3.85)	5.88 (3.32)	<0.001	<0.001	<0.001	0.19
	Co	10.36 (6.47)	3.85 (6.21)	10.27 (6.19)	0.33	0.42	1.00	1.00
	P	0.62	0.03	<0.001	‐	‐	‐	‐
							

### 4.5 Marginal model with generalized estimating equation (GEE) approach

After adjusting demographic characteristic effect, there is a significant relationship between the group variable and self‐efficacy, so that the intervention group had an average of 14.44 more self‐efficacy points than the control group (*p* < 0.001). Also, treatment adherence in the intervention group was significantly higher than the control group (*β* = 6.14, *p* = 0.05). But asthma control was not significant (*β* = −2.29, *p* = 0.064). It should be noted that adjusting the effects of demographic variables has reduced the significance of this variable (Table 6).

**TABLE 6:** Parameters estimation of intervention variable with marginal model after demographic characteristic adjustment.

Outcome	Coefficient	Std. error	Test‐statistics	*p*‐Value
Self‐efficacy	14.43	1.85	60.76	<0.001
Treatment adherence	6.14	3.25	3.55	0.050
Asthma control	−2.29	1.23	3.43	0.064

*Note*: Represented estimation coefficient after adjustment with sex, education level, family income, father's occupation, mother's occupation and time.

## 5 DISCUSSION

In this study, we investigated the effect of motivational interviewing on adherence and self‐efficacy in adolescents with uncontrolled asthma. The findings showed that there were statistically significant differences between the experimental and control group in the mean of adherence and self‐efficacy scores at 2 weeks and 3 months after the intervention. In motivational interviewing, health information is shared in a manner that increases the chance that a patient hears, understands and finally accepts the information. This can be done using clear and understandable language and reflections empathizing with and reducing the patient's concerns. The motivational interviewing uses the ‘encouragement and threat process’ to provide patients’ feedback about their own health (Borrelli et al., 2007).

Reviewing the literatures, we found that although several studies have been done about the effect of motivational interviewing on the adherence and behavioural changes of patients with disorders including psychiatric, blood pressure and diabetes (Ellingson et al., 2019; Fortune et al., 2018; Freira et al., 2017; Ma et al., 2014; Navidian et al., 2017), but few studies have been conducted on the effect of motivational interviewing on adherence and self‐efficacy of patients with asthma.

Miller & Rose mentioned that the theory of motivational interviewing emphasizes two active components underlying the change process in motivational interviewing which can lead to strengthen self‐efficacy. A relational component related to empathy and interpersonal morale and a technical component that involves motivating and empowering the clients, discussion changes (expressions of their desire, ability, reasons and need for change). In addition to therapist attitudes considered for motivational interviewing style and consistency (unconditional positive attention, acceptance, empathy, genuine interest and intimacy, avoidance of coercion), some specific techniques such as strategies to elicit and enhance self‐efficacy have also been considered (Miller & Rose, 2009, 2015).

The result of a recent study by Barikani et al. to investigate the effect of motivational interviewing on self‐efficacy, beliefs about medicines and medication adherence among adolescents with asthma, that was conducted on 52 adolescents with asthma in 2021 showed that the difference between the mean scores in medication adherence, beliefs about medicines and self‐efficacy in the post‐test between the two groups were significant, which is consistent with the results of our study (Barikani et al., 2021). Schmelling et al. conducted another study aimed at changing the attitudes towards medication adherence to asthma on 25 adults with asthma, the results showed a significant difference in the attitudes towards drugs after the intervention. In their study, changes in the attitude of patients towards the drug have been measured, but whether this change in attitude has improved the adherence of individuals has not been studied; however, it shows the positive effect of motivational interviewing, so their findings were consistent with the results of our study (Schmaling et al., 2001). Also, Lavoie et al. performed a study on 54 patients with asthma, which their results were similar to those of the present study achieved within a 3‐month follow‐up in both adherence and self‐efficacy dimensions even within 1 year after the intervention (Lavoie et al., 2014).

Riekert et al. in 2011 investigated the effect of motivational interviewing on adherence of 37 adolescents with asthma (aged 10–15 years old) and their caregivers, and showed that the motivational interviewing increased the motivation of adolescents for treatment, but no significant difference was found in the drug adherence score of the adolescents which is not consistent with the present study. However, in our study, therapeutic adherence was examined and not drug adherence (Riekert et al., 2011). The purpose of the intervention and the target group in the study of Riekert is similar to that of the present study, but their results are not consistent with our study in terms of adherence. It may be because our focus was on adolescents rather than caregivers.

### 5.1 Limitations

There were some limitations in this study. First, there were only two clinics for treatment of adolescents with asthma which we could recruit participants and made fewer samples available for inclusion in the study. Second, patients who were not willing to participate in the study were likely to have low adherence, and this may have led to conduct a study on patients with better adherence. Third, the control group used in this study was standard of care. While a more rigorous control group would have been an attention control group, this was not feasible given the recruitment strategy. Finally, in this study the sample size was small and the treatment effects were only studied at immediate post‐test and 3 months later (the goal was to test the short‐term impact of the intervention). So, it is recommended that the intervention be performed with a larger sample size and longer follow‐up time.

## 6 CONCLUSION

The results presented in this study showed that motivational interviewing is beneficial for adolescents with asthma, and the effects were sustained for 3 months following the intervention. In general, the analysis in this study showed a significant effect of motivational interviewing on adolescents' treatment adherence and self‐efficacy in asthma management and treatment. Therefore, based on the results, some recommendations such as holding short courses of motivational interviewing at appropriate intervals for adolescents can be made for the future.

## AUTHORS’ CONTRIBUTIONS

FT and AN were involved in forming the idea and designing the study, article preparation. AN was responsible for critical revision and final approval of the manuscript. SN was responsible for educational sessions design and data collection. FS conducted and performed data analysis and provided final results.

## CONFLICT OF INTEREST STATEMENT

None declared.

## FUNDING INFORMATION

This research received no specific grant from any funding agency in the public, commercial or not‐for‐profit sectors.

## RESEARCH ETHICS COMMITTEE APPROVAL

This study was approved by the university research ethics committee with the code of ethics of IR.BUMS.REC.1395.179 and the number of IRCT at the National Registry of Clinical Trials is IRCT20170902036026N3.

## PATIENT CONSENT

Participants and their parents were informed about the study, and then they signed the consent form.

## Data Availability

Data available on request from the authors

## References

[nop21679-bib-0001] Al Kindi, Z. , McCabe, C. , & Mc Cann, M. (2021). School nurses' available education to manage children with asthma at schools: A scoping review. Journal of Pediatric Nursing, 60, 46–57. 10.1016/j.pedn.2021.01.027 33610087

[nop21679-bib-0002] Ayala, G. X. , Yeatts, K. , & Carpenter, D. M. (2009). Brief report: Factors associated with asthma management self‐efficacy among 7th and 8th grade students. Journal of Pediatric Psychology, 34(8), 862–868. 10.1093/jpepsy/jsn134 19213736 PMC2734127

[nop21679-bib-0003] Barikani, A. , Negarandeh, R. , Moin, M. , & Fazlollahi, M. R. (2021). The impact of motivational interview on self‐efficacy, beliefs about medicines and medication adherence among adolescents with asthma: A randomized controlled trial. Journal of Pediatric Nursing, 60, 116–122. 10.1016/j.pedn.2021.04.020 33932626

[nop21679-bib-0004] Bektas, İ. , Kudubeş, A. A. , Ayar, D. , & Bektas, M. (2021). Predicting the healthy lifestyle behaviors of Turkish adolescents based on their health literacy and self‐efficacy levels. Journal of Pediatric Nursing, 59, e20–e25. 10.1016/j.pedn.2021.01.016 33589289

[nop21679-bib-0005] Bender, B. G. (2016). Nonadherence to asthma treatment: Getting unstuck. The Journal of Allergy and Clinical Immunology: In Practice, 4(5), 849–851. 10.1016/j.jaip.2016.07.007 27587318

[nop21679-bib-0006] Borrelli, B. , Riekert, K. A. , Weinstein, A. , & Rathier, L. (2007). Brief motivational interviewing as a clinical strategy to promote asthma medication adherence. Journal of Allergy and Clinical Immunology, 120(5), 1023–1030. 10.1016/j.jaci.2007.08.017 17904625

[nop21679-bib-0007] Celano, M. , Robert, J. G. , Keith, M. P. , & Robin, Z. (1998). Treatment adherence among low‐income children with asthma. Journal of Pediatric Psychology, 23(6), 345–349. 10.1093/jpepsy/23.6.345 9824922

[nop21679-bib-0008] Chiu, K.‐C. , Boonsawat, W. , Cho, S.‐H. , Cho, Y. , Hsu, J.‐Y. , Liam, C.‐K. , Muttalif, A. R. , Nguyen, H. D. , Nguyen, V. N. , Wang, C. , & Kwon, N. (2014). Patients' beliefs and behaviors related to treatment adherence in patients with asthma requiring maintenance treatment in Asia. Journal of Asthma, 51(6), 652–659. 10.3109/02770903.2014.898772 PMC4133971 24580369

[nop21679-bib-0009] Dashti, S. , Shahmari, M. , Mirzaaghazadeh, A. , & Mirzaaghazadeh, M. (2016). Effect of foot reflexology and olive oil foot Massageon asthma control. Global Journal of Health Science, 8(12), 53–59. 10.5539/gjhs.v8n12p53

[nop21679-bib-0010] Ellingson, L. , Lansing, J. , DeShaw, K. , Peyer, K. , Bai, Y. , Perez, M. , Phillips, L. A. , & Welk, G. (2019). Evaluating motivational interviewing and habit formation to enhance the effect of activity trackers on healthy Adults' activity levels: Randomized intervention. JMIR mHealth and uHealth, 7(2), e10988. 10.2196/10988 30762582 PMC6393778

[nop21679-bib-0011] Faraji, S. , Valizadeh, S. , Sharifi, A. , Shahbazi, S. , & Ghojazadeh, M. (2020). The effectiveness of telegram‐based virtual education versus in‐person education on the quality of life in adolescents with moderate‐to‐severe asthma: A pilot randomized controlled trial. Nursing Open, 7(6), 1691–1697. 10.1002/nop2.552 33072352 PMC7544889

[nop21679-bib-0012] Fortune, J. , Breckon, J. , Norris, M. , Eva, G. , & Frater, T. (2018). Motivational interviewing training for physiotherapy and occupational therapy students: Effect on confidence, knowledge and skills. Patient Education and Counseling, 102, 694–700. 10.1016/j.pec.2018.11.014 30482468

[nop21679-bib-0013] Fortune, J. , Breckon, J. , Norris, M. , Eva, G. , & Frater, T. (2019). Motivational interviewing training for physiotherapy and occupational therapy students: Effect on confidence, knowledge and skills. Patient Education and Counseling, 102(4), 694–700. 10.1016/j.pec.2018.11.014 30482468

[nop21679-bib-0014] Freira, S. , Lemos, M. S. , Williams, G. , Ribeiro, M. , Pena, F. , & Do Céu Machado, M. (2017). Effect of motivational interviewing on depression scale scores of adolescents with obesity and overweight. Psychiatry Research, 252, 340–345. 10.1016/j.psychres.2017.03.020 28327447

[nop21679-bib-0015] Gomes, A. L. A. , Lima, K. F. , Mendes, E. R. D. R. , Joventino, E. S. , Martins, M. C. , Almeida, P. C. D. , & Ximenes, L. B. (2017). Association of self‐efficacy of parents/caregivers with childhood asthma control parameters. Revista da Escola de Enfermagem da USP, 51, e03282. 10.1590/S1980-220X2017008003282 29562048

[nop21679-bib-0016] Holley, S. , Knibb, R. , Latter, S. , Liossi, C. , Mitchell, F. , Radley, R. , & Roberts, G. (2019). Development and validation of the adolescent asthma self‐efficacy questionnaire (AASEQ). European Respiratory Journal, 54(1), 1801375. 10.1183/13993003.01375-2018 31048348

[nop21679-bib-0017] Jentzsch, N. S. , Silva, G. C. , Mendes, G. M. , Brand, P. L. , & Camargos, P. (2017). Treatment adherence and level of control in moderate persistent asthma in children and adolescents treated with fluticasone and salmeterol. Jornal de Pediatria, 95, 69–75. 10.1016/j.jped.2017.10.008 29274305

[nop21679-bib-0018] Kaplan, A. , & Price, D. (2020). Treatment adherence in adolescents with asthma. Journal of Asthma and Allergy, 13, 39–49. 10.2147/JAA.S233268 32021311 PMC6969681

[nop21679-bib-0020] Lavoie, K. L. , Moullec, G. , Lemiere, C. , Blais, L. , Labrecque, M. , Beauchesne, M.‐F. , Pepin, V. , Cartier, A. , & Bacon, S. L. (2014). Efficacy of brief motivational interviewing to improve adherence to inhaled corticosteroids among adult asthmatics: Results from a randomized controlled pilot feasibility trial. Patient Preference and Adherence, 8, 1555–1569. 10.2147/PPA.S66966 25422587 PMC4231985

[nop21679-bib-0021] Ma, C. , Zhou, Y. , Zhou, W. , & Huang, C. (2014). Evaluation of the effect of motivational interviewing counselling on hypertension care. Patient Education and Counseling, 95(2), 231–237. 10.1016/j.pec.2014.01.011 24530144

[nop21679-bib-0022] Martin, M. A. , Catrambone, C. D. , Kee, R. A. , Evans, A. T. , Sharp, L. K. , Lyttle, C. , Rucker‐Whitaker, C. , Weiss, K. B. , Shannon, J. J. , & Team, C. I. t. R. A. H. E. I . (2009). Improving asthma self‐efficacy: Developing and testing a pilot community‐based asthma intervention for African American adults. Journal of Allergy and Clinical Immunology, 123(1), 153–159. e153. 10.1016/j.jaci.2008.10.057 19130936 PMC2675162

[nop21679-bib-0023] Miller, W. R. , & Rose, G. (2009). Toward a theory of motivational interviewing. American Psychologist, 64(6), 527–537. 10.1037/a0016830 19739882 PMC2759607

[nop21679-bib-0024] Miller, W. R. , & Rose, G. S. (2015). Motivational interviewing and decisional balance: Contrasting responses to client ambivalence. Behavioral and cognitive. Psychotherapy, 43(2), 129–141. 10.1017/S1352465813000878 24229732

[nop21679-bib-0025] Mirkarimi, K. , Kabir, M. J. , Honarvar, M. R. , Ozouni‐Davaji, R. B. , & Eri, M. (2017). Effect of motivational interviewing on weight efficacy lifestyle among women with overweight and obesity: A randomized controlled trial. Iranian Journal of Medical Sciences, 42(2), 187–193. 28360445 PMC5366367

[nop21679-bib-0026] Morton, K. , Beauchamp, M. , Prothero, A. , Joyce, L. , Saunders, L. , Spencer‐Bowdage, S. , Dancy, B. , & Pedlar, C. (2015). The effectiveness of motivational interviewing for health behaviour change in primary care settings: A systematic review. Health Psychology Review, 9(2), 205–223. 10.1080/17437199.2014.882006 26209209

[nop21679-bib-0027] Myers, J. M. B. , Schauberger, E. , He, H. , Martin, L. J. , Kroner, J. , Hill, G. M. , Ryan, P. H. , GK, L. M. , Bernstein, D. I. , Lockey, J. E. , Arshad, S. H. , Kurukulaaratchy, R. , & Khurana Hershey, G. K. (2018). A pediatric asthma risk score to better predict asthma development in young children. The Journal of Allergy and Clinical Immunology, 143, 1803–1810.e2. 10.1016/j.jaci.2018.09.037 30554722 PMC6504569

[nop21679-bib-0028] Navidian, A. , Mobaraki, H. , & Shakiba, M. (2017). The effect of education through motivational interviewing compared with conventional education on self‐care behaviors in heart failure patients with depression. Patient Education and Counseling, 100(8), 1499–1504. 10.1016/j.pec.2017.02.023 28262273

[nop21679-bib-0029] Riekert, K. A. , Borrelli, B. , Bilderback, A. , & Rand, C. S. (2011). The development of a motivational interviewing intervention to promote medication adherence among inner‐city, African‐American adolescents with asthma. Patient Education and Counseling, 82(1), 117–122. 10.1016/j.pec.2010.03.005 20371158 PMC2937081

[nop21679-bib-0030] Schmaling, K. B. , Blume, A. W. , & Afari, N. (2001). A randomized controlled pilot study of motivational interviewing to change attitudes about adherence to medications for asthma. Journal of Clinical Psychology in Medical Settings, 8(3), 167–172. 10.1023/A:1011365519345

[nop21679-bib-0031] Valizadeh, L. , Zarei, S. , Zamanazadeh, V. , Bilan, N. , Nasiri, K. , & Howard, F. (2014). The effects of Triggers' modifying on adolescent self‐efficacy with asthma: A randomized controlled clinical trial. Journal of Caring Sciences, 3(2), 121–129. 10.5681/jcs.2014.013 25276755 PMC4134172

[nop21679-bib-0032] Zarei, A. R. , Jahanpour, F. , Alhani, F. , Razazan, N. , & Ostovar, A. (2014). The impact of multimedia education on knowledge and self‐efficacy among parents of children with asthma: A randomized clinical trial. Journal of Caring Sciences, 3(3), 185–192. 10.5681/jcs.2014.020 25276762 PMC4171813

